# Simultaneous detection of dental caries and fissure sealant in intraoral photos by deep learning: a pilot study

**DOI:** 10.1186/s12903-024-04254-1

**Published:** 2024-05-12

**Authors:** Yanshan Xiong, Hongyuan Zhang, Shiyong Zhou, Minhua Lu, Jiahui Huang, Qiangtai Huang, Bingsheng Huang, Jiangfeng Ding

**Affiliations:** 1 Department of Endodontics, Shenzhen Stomatology Hospital, Shenzhen, Guangdong China; 2 Medical AI Lab, School of Biomedical Engineering, Medical School, Shenzhen University, Shenzhen, Guangdong China; 3 Guangdong Key Laboratory for Biomedical Measurements and Ultrasound Imaging, School of Biomedical Engineering, Medical School, Shenzhen University, Shenzhen, Guangdong China; 4 Department of Pediatric Stomatology, Shenzhen Stomatology Hospital, Shenzhen, Guangdong China

**Keywords:** Dental caries, Fissure sealant, Artificial intelligence, Deep learning

## Abstract

**Background:**

Deep learning, as an artificial intelligence method has been proved to be powerful in analyzing images. The purpose of this study is to construct a deep learning-based model (ToothNet) for the simultaneous detection of dental caries and fissure sealants in intraoral photos.

**Methods:**

A total of 1020 intraoral photos were collected from 762 volunteers. Teeth, caries and sealants were annotated by two endodontists using the LabelMe tool. ToothNet was developed by modifying the YOLOX framework for simultaneous detection of caries and fissure sealants. The area under curve (AUC) in the receiver operating characteristic curve (ROC) and free-response ROC (FROC) curves were used to evaluate model performance in the following aspects: (i) classification accuracy of detecting dental caries and fissure sealants from a photograph (image-level); and (ii) localization accuracy of the locations of predicted dental caries and fissure sealants (tooth-level). The performance of ToothNet and dentist with 1year of experience (1-year dentist) were compared at tooth-level and image-level using Wilcoxon test and DeLong test.

**Results:**

At the image level, ToothNet achieved an AUC of 0.925 (95% CI, 0.880–0.958) for caries detection and 0.902 (95% CI, 0.853–0.940) for sealant detection. At the tooth level, with a confidence threshold of 0.5, the sensitivity, precision, and F1-score for caries detection were 0.807, 0.814, and 0.810, respectively. For fissure sealant detection, the values were 0.714, 0.750, and 0.731. Compared with ToothNet, the 1-year dentist had a lower F1 value (0.599, *p* < 0.0001) and AUC (0.749, *p* < 0.0001) in caries detection, and a lower F1 value (0.727, *p* = 0.023) and similar AUC (0.829, *p* = 0.154) in sealant detection.

**Conclusions:**

The proposed deep learning model achieved multi-task simultaneous detection in intraoral photos and showed good performance in the detection of dental caries and fissure sealants. Compared with 1-year dentist, the model has advantages in caries detection and is equivalent in fissure sealants detection.

## Background

Dental caries is a major disease that impacts human health and quality of life, affecting 60–90% of school-aged children and the vast majority of adults in most industrialized countries [1]. If not treated in time, caries can further develop into pulp-periapical disease and even lead to tooth loss. Pit and fissure sealing is internationally recognized as an effective method for preventing pit and fissure caries [2]. Regular oral examination and fissure sealing are important strategies for caries prevention [3]. However, in many countries, including China, medical resources are insufficient or unevenly distributed, so it is difficult to increase the use of face-to-face consultations for routine monitoring, especially in remote areas and for special groups. In addition, these face-to-face consultations have limited use for mass screening or responses to public health emergencies. Therefore, telemedicine currently has great application value.

In recent years, image processing technology based on artificial intelligence has made rapid progress and has been widely used in medical image analysis. Deep learning (DL) based on convolution neural networks (CNNs), as an artificial intelligence method, has been proven to be powerful in analysing images [4]. In the dental field, deep learning is mainly used to analyse the results of radiological examinations for orthodontics and detection of caries, periapical disease, and periodontitis [5]. For caries detection, these examinations mainly include apical radiographs, bitewing radiographs and images generated by newer caries detection techniques, such as near-infrared transilluminated imaging and optical coherence tomography [6–12]. Deep learning models already have demonstrated good detection performance in analysing these examination results. However, although these methods can assist in the detection of dental caries, they require professional equipment and doctors and therefore cannot meet the needs of telemedicine.

With the rapid development of handheld image acquisition technology, image acquisition is convenient and fast, and the image quality tends to be high. The number of studies on the automatic detection of handheld images with deep learning, such as the automatic detection of dental caries, gingivitis, pit and fissure sealants, and restorations, has gradually increased [13–16]. Currently, most studies have collected processed or standardized high-quality professional photos and have only included data that serves a certain purpose. In real-life scenarios, a lack of access to high-quality photos may lead to model performance degradation. At the same time, oral problems are diverse, and there may be multiple problems with one tooth. For example, sealants or fillings may be excluded from datasets in studies of dental caries. Such a model with a specific detection target cannot identify excluded dental diseases unless additionally trained. Therefore, it is necessary to develop a detection model that can perform multiple tasks and be used by nonprofessional people in daily life scenarios.

In this study, we developed a deep learning-based intelligent detection model (ToothNet) for the simultaneous detection of caries and fissure sealants in intraoral occlusal photos and evaluated the model performance. We preliminarily verified the clinical feasibility of the model by comparing it with the diagnostic results of dentists. Our hypothesis was that the performance of ToothNet is comparable to that of dentist.

## Methods

### Data acquisition

Our study was approved by the Medical Ethics Committee of Shenzhen Stomatological Hospital, and was performed in accordance with the Declaration of Helsinki. A total of 1020 intraoral panoramic maxillary/mandibular occlusal photos were collected from 762 volunteers. Volunteers range in age from 4 to 55 years old. All the data were acquired at Shenzhen Stomatological Hospital between October 2021 and December 2022, with the informed consent of volunteers or their parents. The photos were taken by four common cameras (Canon EOS 6D2, NIKON D80, iPhone XS, iPhone 11 Pro Max). When the photos were taken, the volunteer opened their mouth wide enough to expose as much of the full dentition as possible. The parameters of each device were not uniformly set, and the photos were all taken in the automatic mode. No specific inclusion or exclusion criteria (such as brightness, resolution, shooting angle, etc.) were applied to force the established DL model to adapt as much as possible, as is required in real-life scenarios.

### Image annotation

**Fig. 1:**
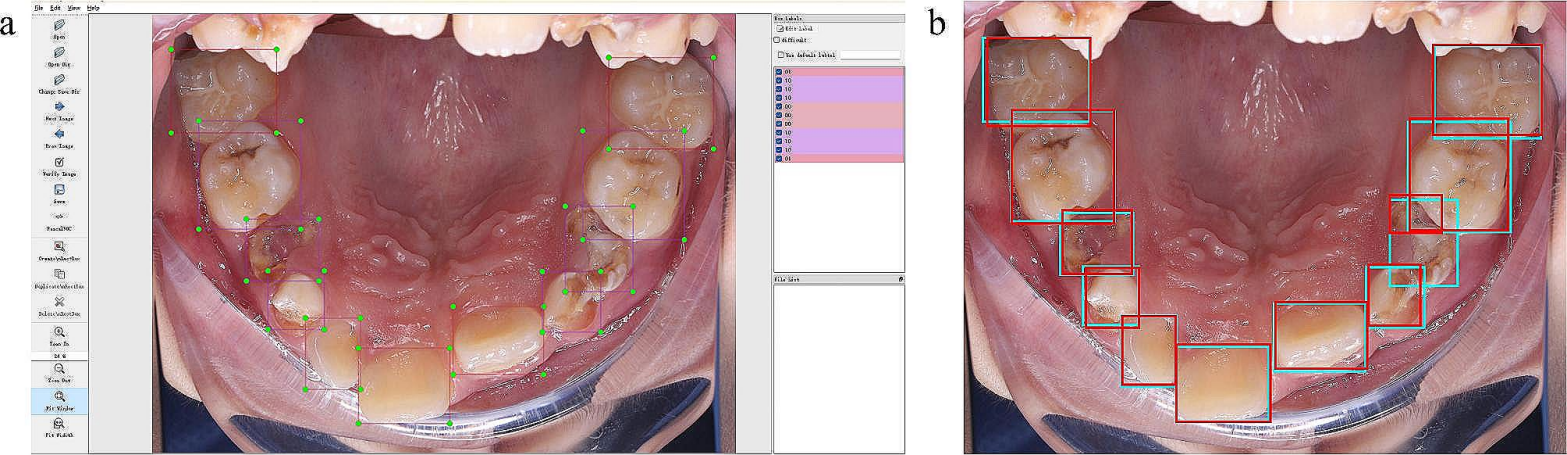
Example of image annotation (**a**). Example of single tooth localization visualization; blue and red bounding boxes denote annotation boxes and model prediction, respectively (**b**)

First, each tooth was labelled using the LabelMe tool (version 5.0.0; https://github.com/wkentaro/labelme) by two endodontists with five years of experience. The labels appeared as multiple independent or partially overlapping rectangles. Then, referring to the International Caries Detection and Assessment System (ICDAS) and caries assessment spectrum and treatment (CAST), the caries lesions on a single tooth and the retention of fissure sealants were annotated according to the results of the inspection [17, 18]. Each label consisted of two digits: the tens digits was set to “1” or “0” to indicate the presence or absence of caries (ICDAS code 3 or greater), respectively, and the ones digits was set to “1” or “0” to indicate the presence or absence of sealants. An annotation example is shown in Fig. 1a.

Prior to formal labelling, a chief physician with fifteen years of experience guided two endodontists in studying and labelling 100 photos that were not included in this study. The consistency of the annotations made by the two endodontists was assessed using a consistency test, with the requirement that the Kappa coefficient should range from 0.8 to 1.0.

### Dataset partition

In our study, only one panoramic maxillary or mandibular occlusion photograph was included for each patient to ensure the independence of the image data. The dataset, comprising 1020 oral images, was randomly divided into training, validation and test sets, which contained 720, 100 and 200 photos, respectively. The three data sets were mutually exclusive, and since only one photo was taken of each patient’s maxilla/mandible, it was impossible for the same tooth from the same patient to appear in two photos. We separately counted the number of teeth, caries, and fissure sealants in the different datasets. The specific details are presented in Table 1.

**Table 1:** Details of the labels in different datasets

	photos	teeth	caries	no caries	complete or partial sealant	no sealant
training set	720	9030	1995	7035	328	8702
validation set	100	1263	271	992	39	1224
test set	200	2553	622	1931	84	2469

### DL model architecture

**Fig. 2:**
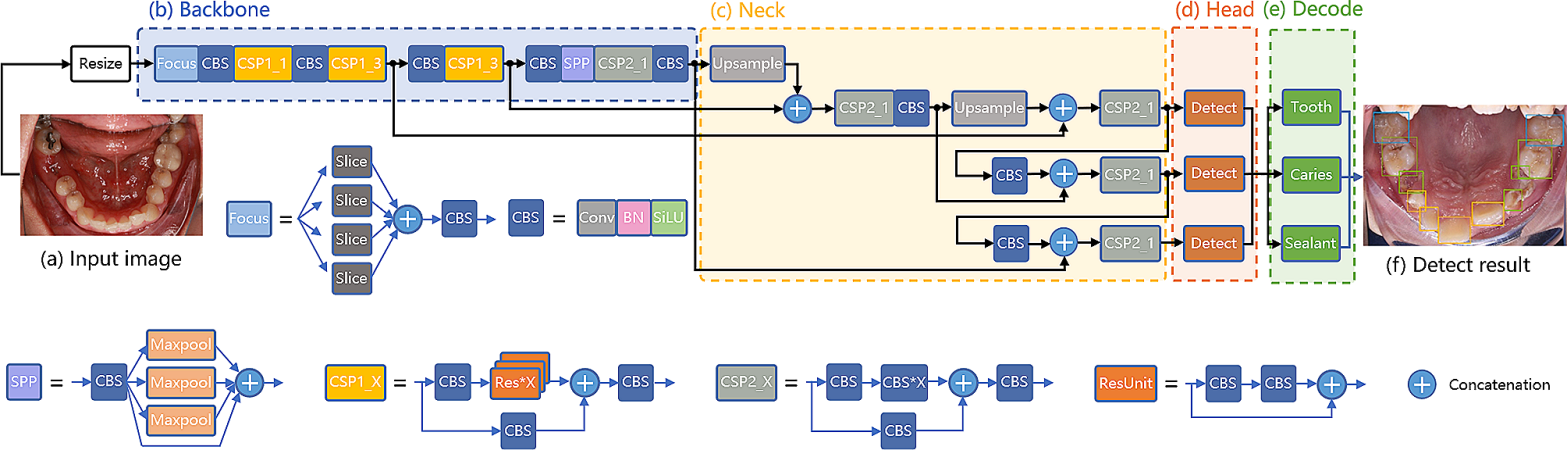
Model architecture overview. Our model consists of three parts: (**b**) The backbone for extracting features; (**c**) the neck for feature fusion; and (**d**) the head for detection and classification of features at different scales. Given an intraoral photo (**a**) as the input, the outputs of different scales are finally decoded (**e**) to output each tooth, caries and sealant detection result (**f**)

Our study consisted of three crucial tasks: tooth localization, caries detection, and fissure sealant detection. Tooth localization was performed to ensure that model detection was performed within the region of interest (i.e., teeth). Tooth localization was conducted as an essential preprocessing step to define the region of interest (i.e., the teeth) within the intraoral images, facilitating accurate tooth numbering and subsequent model detections for caries and fissure sealants. This ensured precision in identifying dental structures and optimized the reliability of our study’s results. To tackle this challenge, we introduced ToothNet, a single multi-task learning (MTL) convolutional neural network that can perform all three tasks. The architecture of ToothNet is illustrated in Fig. 2: an intraoral image (Fig. 2a) serves as the input, and the model outputs the location and classification probabilities of detected teeth, caries, and sealants (Fig. 2f). We enhanced the YOLOX model, an anchor-free detection framework, by extending its detection head to include three classification outputs: tooth classification, caries classification, and sealant classification, along with a detection box regression head. The tooth classification head utilizes a convolutional layer with an output channel of 1 to distinguish teeth from background regions. Similarly, the caries and sealant classification heads also employ convolutional layers with an output channel of 1 to classify instances of teeth. The detection box regression head utilizes a convolutional layer with a 4-channel output to predict the bounding boxes (x, y, w, h) of teeth. These two instance classification head outputs correspond to the two oral health analysis tasks, e.g. caries detection, and fissure sealant detection, included in our model. Figure 2 also presents an example of ToothNet’s outputs on test images. The entire model was optimized in an end-to-end manner and executed simultaneously to produce results for all three tasks. As open science, the source code is available at https://github.com/MedcAILab/ToothNet.

### DL model training strategy

The intersection over union (IoU) loss and binary cross-entropy (BCE) loss are commonly used in object detection tasks [19, 20]. The IoU loss measures the accuracy of bounding box regression by evaluating the overlap between predicted and ground truth bounding boxes. On the other hand, BCE loss is used for classification tasks, assessing the accuracy of class predictions. Following previous detection works [21, 22], we set the weighting between the IoU loss and BCE loss to 1:1. This configuration ensures that the model treats classification and bounding box detection equally during training, allowing it to learn effectively from both types of information. Besides, to facilitate neural network input, each image was uniformly resized to 640 by 640 pixels, maintaining the original aspect ratio.

To enhance the diversity of our training dataset and improve model robustness [23, 24], we also employed an extensive data augmentation strategy on the input images, including random shifting, cropping, rotation, scaling, and colour channel shifting. Specifically, images were randomly shifted horizontally and vertically with a 0.5 probability, rotated within a range of -20° to 20°, and scaled between 0.75 and 1.25. Besides, random changes in hue, saturation, and exposure were introduced using uniform random values sampled from the range of (-1, 1) to augment colour variations in the images. In addition, the model weights were initialized with pretrained weights on a public COCO dataset to expedite the training process.

The ToothNet model we developed used the PyTorch framework (version 1.10.1; https://pytorch.org/). The model was trained on the Ubuntu 16.04 operating system with an NVIDIA TITIAN RTX GPU and Intel Xeon E5-2650 2.30 GHz CPU. The model parameters were updated using the Stochastic Gradient Descent (SGD) optimizer, the learning rate was set to 1e-3, and the momentum was set to 0.99. We performed early stopping mechanism on the validation loss with a patience of 10 epochs to avoid over-fitting. Our deep learning model had approximately 2.5 million parameters in total. Utilizing the early stopping mechanism, the training process concluded after approximately 10 h, equivalent to around 150 epochs.

### Model evaluation

For single tooth detection, sensitivity, precision and the rate of false positives per image were applied to evaluate model performance under different intersection-over-union (IoU) thresholds.

To evaluate our caries and sealant detection model, we considered two aspects: (i) classification performance in determining the presence or absence of caries or sealant at the image level, and (ii) localization performance in identifying particular regions with caries or sealant in the images at the tooth level.

In terms of classification performance at the image level, we utilized receiver operating characteristic (ROC) analysis and calculated the area under the curve (AUC) and corresponding 95% confidence interval (95% CI), accuracy (ACC), sensitivity (SEN), specificity (SPE), positive predictive value (PPV), and negative predictive value (NPV). All metrics were calculated based on the optimal cut-off value that maximized the Youden index [25].

In terms of localization performance at the tooth level, we measured the free-response ROC (FROC) curve, which plots the bounding box true positive rate, or sensitivity (SEN) vs. the average number of false positive (FP) boxes per image with different thresholds for box probabilities. In addition to SEN and FP, we also considered precision (PRE), average precision (AP), and F1-score as crucial evaluation metrics. The F1-score combined precision and sensitivity to provide a single score. It ranges from 0 to 1, where a higher F1-score indicates better model performance. A perfect model has a TPR of 1.0 at an FP of 0.0, indicating that the model detects all the dental caries and fissure sealants without any false-positive predictions while maintaining high precision and F1-score.

In the detection task, the sensitivity, precision, and F1-score can be calculated as follows:

1 $$sensitivity= \frac{TP}{TP+FN}$$

2 $$precision= \frac{TP}{TP+FP}$$

3 $${F}_{1}-score= \frac{2*sensitivity*precision}{\left(sensitivity+precision\right)}$$

where TP, FP and FN are the abbreviations of true positive, false positive and false negative, respectively.

### Performance comparation between ToothNet and 1-year dentist

To verify the clinical feasibility, we invited two dentists, each with one year of clinical experience, to label the caries and fissure sealants in the test set. Prior to formal annotation, we provided them with training in visual diagnosis of intraoral photos and the use of annotation software. Subsequently, we selected photos that were not part of the test set to evaluate their consistency. Only when the consistency meets the standards can they be formally annotated. We then compared the diagnostic results of the 1-year dentists with those of ToothNet. The methods and performance metrics used have been explained in the preceding section.

### Statistical analysis

All analyses were conducted using MedCalc statistical software (version 20.0.9.0; https://www.medcalc.org/), and Python (version 3.10.9; https://www.python.org/). Wilcoxon test was used to compare the F1-scores of the ToothNet and 1-year dentist’s diagnostic results at tooth-level. DeLong test was utilized to compare the AUCs of the ToothNet and 1-year dentist’s diagnostic results at the image level. All tests were two-sided, and *P* < 0.05 was considered statistically significant.

## Results

### Single tooth localization performance

Table 2 shows the performance of the model for single tooth localization. When the IoU was set at 0.5, the sensitivity, accuracy and false positive per image of the model for tooth detection were 0.994, 0.998 and 0.08, respectively. We selected this threshold for subsequent analyses of caries and sealant detection. Figure 1b shows an example of tooth localization.

**Table 2:** Tooth location performance

	SEN	PRE	FP / image	TP	FP	FN
IoU0.25	0.995	0.999	0.065	2540	1	13
IoU0.50	0.994	0.998	0.080	2537	4	16
IoU0.75	0.975	0.979	0.325	2488	53	65

* TP = True Positive; FP = False Positive; FN = False Negative

### Dental caries and fissure sealant detection performance of ToothNet

**Fig. 3:**
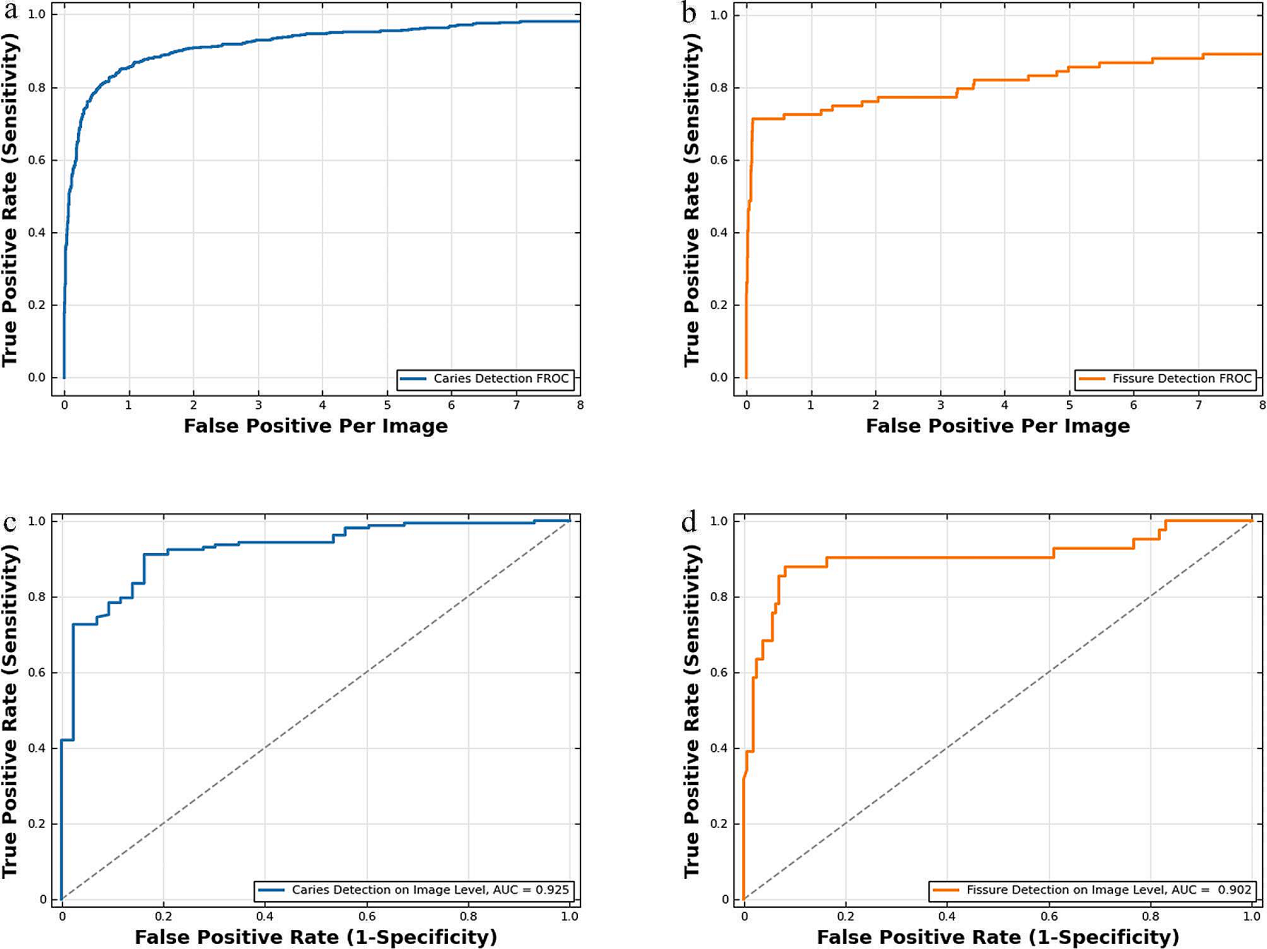
FROC curves of tooth level caries detection (**a**). FROC curves of tooth level sealant detection (**b**). ROC curves of image level caries detection (**c**). ROC curves of image level sealant detection (**d**)

The tooth level performance of the deep learning model in the test set is shown in Table 3; Fig. 3. At a confidence threshold of 0.5, the sensitivity and precision of the model were 80.7% and 81.4% for caries detection and 71.4% and 75.0% for sealant detection, respectively (Table 3). Besides, our evaluation revealed AP scores of 0.785 for caries detection and 0.635 for sealant detection. The FROC curves for caries detection and sealant detection are shown in Fig. 3a and b.

The ROC curves for image-level caries detection and sealant detection are shown in Fig. 3c and d. The AUC of the model was 0.925 (95% CI: 0.880–0.958) for caries detection and 0.902 (95% CI: 0.853–0.940) for sealant detection. At the optimal threshold of the ROC curve, the sensitivity and specificity of the model were 91.1% and 83.7% for caries detection and 87.8% and 91.8% for sealant detection, respectively (Table 4).

**Fig. 4:**
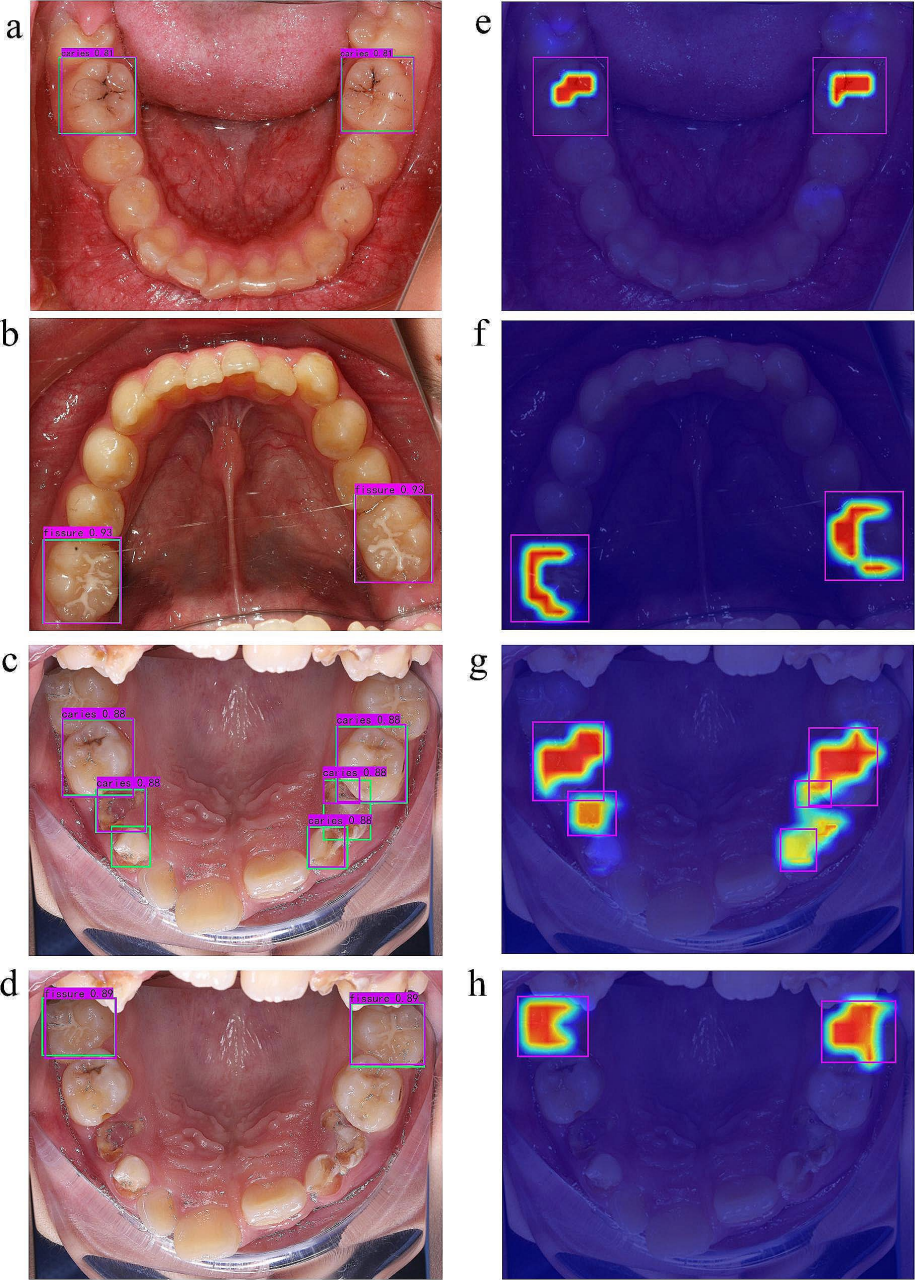
Visualization of caries and sealant detection results. In panels **a, b, c** and **d**, the green box represents the real bounding box, and the purple box is the model prediction bounding box; figures **e, f, g** and **h** are the corresponding attention area heatmaps, and red indicates a high level of attention

An example of the model output visualization is shown in Fig. 2f. To show the results of different tasks more clearly, we separately show their prediction results and corresponding visual heatmaps, as shown in Fig. 4.

### Performance comparation between ToothNet and 1-year dentist

Table 3 presents the performance evaluation of the 1-year dentist at the tooth-level for caries and fissure sealant detection. In caries detection, the 1-year dentist exhibited a sensitivity of 0.428 and a precision of 1.000, resulting in an F1-score of 0.599. For sealant detection, the 1-year dentist demonstrated a sensitivity of 0.517 and a precision of 1.000, yielding an F1-score of 0.727. Based on the comprehensive metric of F1 score, ToothNet outperformed the 1-year dentist in caries detection (*p* < 0.0001) and sealant detection (*p* = 0.023).

As demonstrated in Table 4, at the image level, the 1-year dentist achieved an AUC of 0.794 (95% CI: 0.683–0.807) for caries detection, which was significantly lower than that of ToothNet (*p* < 0.0001). For fissure sealants detection, the AUC was 0.829 (95% CI: 0.769–0.878), which was comparable to that of ToothNet (*p* = 0.154).

**Table 3:** The performance of *ToothNet and 1-year dentist* on the tooth-level

	Methods	AP	SEN	PRE	F1-score	FP / image	*p* value
caries detection	ToothNet	0.785	0.807	0.814	0.810	0.600	*p* < 0.0001
	dentist	/	0.428	1.000	0.599	1.780	
sealer detection	ToothNet	0.635	0.714	0.750	0.731	0.120	*p* = 0.0230
	dentist	/	0.517	1.000	0.727	0.180	

* The *p*-values were obtained through Wilcoxon tests conducted on the Fl-score metric

**Table 4:** The performance of *ToothNet and 1-year dentist* on the image-level

	Methods	AUC [95% CI]	ACC	SEN	SPE	PPV	NPV	*p* value
caries detection	ToothNet	0.925 [0.880–0.958]	0.895	0.911	0.837	0.953	0.720	*p* < 0.0001
	dentist	0.749 [0.683–0.807]	0.685	0.637	0.860	0.943	0.394	
sealer detection	ToothNet	0.902 [0.853–0.940]	0.910	0.878	0.918	0.735	0.967	*p* = 0.1545
	dentist	0.829 [0.769–0.878]	0.915	0.683	0.975	0.875	0.923	

* The *p*-values were obtained through DeLong tests conducted on the AUC metric

## Discussion

At present, clinical visuo-tactile or visual examinations are still the standard for the diagnosis of caries [26]. However, photographic assessment methods with intraoral digital photographs by dentists are comparable to visual examinations [27]. This shows the feasibility of remote diagnosis of oral diseases based on clinical visual examinations. In our study, we used expert annotation as the ‘gold standard’; two experienced endodontists who passed the consistency test annotated the images, thus ensuring the accuracy and reliability of the annotation. Since our detection targets were teeth with caries and sealants, we also trained the DL model to localize individual teeth on intraoral photos. In this way, the output images of the model can also more comprehensively and intuitively display the overall situation of the dentition. The DL model had a high sensitivity of 99.4% and accuracy of 99.8% in single tooth segmentation, ensuring its reliability for caries and sealant detection tasks.

For dental caries detection, most previous studies collected high-quality images of a single tooth and then classified the images to achieve detection [12, 28, 29]. However, this image acquisition and detection method is not suitable for real-life scenarios. In our study, the photos we acquired were panoramic maxillary/mandibular occlusal photos, which can display the most important information of the oral cavity with only two images. The acquisition process was simple and easy for non-professionals to learn and use in daily life scenarios. In terms of the detection method, we conducted simultaneous detection, localization, and classification with a single photo. There are two previous studies that are particularly related to our research. Zhang et al. [30] obtained partial oral photos using consumer cameras and trained their model using a hard negative mining algorithm. They reported an imagewise sensitivity of 81.90%, a boxwise sensitivity of 64.60%, and an AUC of 85.65%. Second, ding et al. [31] utilized the YOLOv3 algorithm to detect caries in oral photos captured by mobile phones and achieved a model mean average precision (mAP) of 85.48%. In our study, the DL model ToothNet was developed by modifying the YOLOX framework, which has a sensitivity of 80.7% and an accuracy of 81.4% at the tooth level and an AUC of 92.5% at the image level for caries detection. Distinct from the previous two studies, we detected carious teeth in photos rather than carious lesions, focusing more on the overall condition of a single tooth. In this way, multitask detection of caries and sealant can be performed on one intraoral photo at the same time, which is more in line with clinical inspection and recording habits. In addition, the model can also be used to develop other detection tasks in the future.

With respect to the detection of fissure sealants, there is only one strongly related study. Schlichenrieder et al. [15] used standardized high-quality professional clinical photos, each including only one tooth, and excluded caries, developmental defects, and teeth with restorations. Their purpose was to exclude interfering information to obtain higher model performance. In our study, the AUC, sensitivity and precision of our model for sealant detection reached 90.2%, 74.1% and 71.4%, respectively. Compared with our other two detection tasks, the detection performance of the fissure sealants requires further improvements. For the diversification and authenticity of the data, we did not require photos to contain dental caries or fissure sealants, so the samples with sealants were limited. We may collect relevant data at a later stage to further improve the detection performance.

To preliminarily assess clinical feasibility, we compared the model’s diagnostic results with those of 1-year dentist. The results showed that, for caries detection, although 1-year dentist’s accuracy was high, they had more false negatives and lower sensitivity. Overall, our model exhibited higher F1 scores and AUC compared to them (*p* < 0.05), demonstrating an advantage in caries detection. In terms of application, the model’s primary use is for routine monitoring among non-professionals, so it needs to ensure high sensitivity to screen out suspicious caries. As for sealant detection, 1-year dentist still exhibited high accuracy and lower sensitivity, which may be due to their limited clinical experience and conservative diagnostic tendencies. In particular, they do not diagnose occult caries well. However, in terms of comprehensive metric, the F1 score of the model was slightly better than that of 1-year dentist (*p* < 0.05), while the model’s AUC was not statistically different from that of 1-year dentist (*p* > 0.05), indicating that the model is comparable to 1-year dentist in sealant detection. Nevertheless, due to the limited number and levels of dentists included, further experiments are required to verify the model’s feasibility.

Our study still has some shortcomings that require further research in the future. First, the dataset is relatively limited, especially data containing fissure sealants, which may be one of the reasons why the model was not as good as caries detection in fissure sealant detection. In the future, we will enrich the dataset to further improve model performance and robustness. Second, the detection of occult caries is greatly affected by image quality and shooting light. How to further improve the accuracy of the identification needs further discussion. Third, for the white opaque lesions of early caries, it usually requires continuous air gun drying before detection. It is difficult to achieve this condition in real-life scenarios. We therefore did not make this a requirement when collecting images, so the model was unable to distinguish the first visual changes in enamel. Despite being tested on a diverse dataset comprising multi-devices, varying lighting conditions, and different types of data, our model demonstrated performance on par with that of a doctor with one year of experience. Moving forward, we intend to expand our dataset to encompass even greater diversity and undertake prospective studies in clinical settings to further validate and refine the model’s performance.

## Conclusions

It is feasible to detect caries and fissure sealants from panoramic occlusal photos with methods based on deep learning. The deep learning model constructed in this study can accurately locate teeth and shows good performance in the detection of caries and fissure sealants. Compared with the dentist with 1year of experience, the model has advantages in caries detection and is equivalent in fissure sealants detection. In the future, we may expand the database in more varied real-life scenarios to further improve the performance of the model, with a view to realizing artificially intelligent oral examinations.

## Data Availability

The datasets used and analysed during the current study are available from the corresponding author on reasonable request. The data are not publicly available due to privacy restrictions.

## Abbreviations

ROC: Receiver operating characteristic
FROC: Free-response ROC
DL: Deep learning
CNNs: Convolution neural networks
ICDAS: International Caries Detection and Assessment System
CAST: Caries assessment spectrum and treatment
MTL: Multitask learning
IoU: Intersection over union
AUC: Area under the curve
ACC: Accuracy
SEN: Sensitivity
SPE: Specificity
PPV: Positive predictive value
NPV: Negative predictive value
TPR: True positive rate
FP: False-positive
mAP: Mean average precision
CI: Confidence Interval
